# Machine Learning and Bioinformatics Models to Identify Pathways that Mediate Influences of Welding Fumes on Cancer Progression

**DOI:** 10.1038/s41598-020-57916-9

**Published:** 2020-02-17

**Authors:** Humayan Kabir Rana, Mst. Rashida Akhtar, M. Babul Islam, Mohammad Boshir Ahmed, Pietro Lió, Fazlul Huq, Julian M. W. Quinn, Mohammad Ali Moni

**Affiliations:** 1 Department of Computer Science and Engineering, Green University of Bangladesh, Dhaka, Bangladesh; 2 Department of Computer Science and Engineering, Varendra University, Rajshahi, Bangladesh; 3 Department of Electrical and Electronic Engineering, University of Rajshahi, Rajshahi, Bangladesh; 4 Bio-electronics Materials Laboratory, School of Materials Science and Engineering, Gwangju Institute of Science and Technology, 261 Cheomdan-gwagiro, Buk-gu, Gwangju, 500-712 Republic of Korea; 5 Computer Laboratory, Department of Computer Science and Technology, University of Cambridge, 15 JJ Thomson Avenue, Cambridge, CB3 0FD UK; 6 Discipline of Pathology, School of Medical Sciences, Faculty of Medicine and Health, The University of Sydney, Sydney, Australia; 7 Bone Biology Division, Garvan Institute of Medical Research, Darlinghurst, NSW Australia

**Keywords:** Cancer genomics, Machine learning

## Abstract

Welding generates and releases fumes that are hazardous to human health. Welding fumes (WFs) are a complex mix of metallic oxides, fluorides and silicates that can cause or exacerbate health problems in exposed individuals. In particular, WF inhalation over an extended period carries an increased risk of cancer, but how WFs may influence cancer behaviour or growth is unclear. To address this issue we employed a quantitative analytical framework to identify the gene expression effects of WFs that may affect the subsequent behaviour of the cancers. We examined datasets of transcript analyses made using microarray studies of WF-exposed tissues and of cancers, including datasets from colorectal cancer (CC), prostate cancer (PC), lung cancer (LC) and gastric cancer (GC). We constructed gene-disease association networks, identified signaling and ontological pathways, clustered protein-protein interaction network using multilayer network topology, and analyzed survival function of the significant genes using Cox proportional hazards (Cox PH) model and product-limit (PL) estimator. We observed that WF exposure causes altered expression of many genes (36, 13, 25 and 17 respectively) whose expression are also altered in CC, PC, LC and GC. Gene-disease association networks, signaling and ontological pathways, protein-protein interaction network, and survival functions of the significant genes suggest ways that WFs may influence the progression of CC, PC, LC and GC. This quantitative analytical framework has identified potentially novel mechanisms by which tissue WF exposure may lead to gene expression changes in tissue gene expression that affect cancer behaviour and, thus, cancer progression, growth or establishment.

## Introduction

Welding processes join rigid material pieces (usually metal) at their contact interface by using high temperatures to cause fusion. This process can be hazardous because it exposes the operator to extremely toxic fumes and to radiant energy^1^. The International Agency for Research on Cancer (IARC) has recognized WFs and UV radiation from welding as Group 1 carcinogens^2^. WFs are mainly composed of metallic oxides, silicates and fluorides, including those of magnesium, manganese, zinc, aluminum, beryllium, copper, chromium, cadmium, lead, iron, nickel and vanadium^3^.

Welders inhaling WFs in large quantities over a long period run a significantly elevated risk of developing certain types of cancer^1,2^. These metastatic diseases involve uncontrolled or neoplastic growth of cancer cells that arise after the accumulation of genomic mutations, but other factors with powerful effects on cancer behaviour and growth include genetic factors and environmental factors the suffer is exposed to^4^. Environmental factors include inhaled toxic fumes that affect the lungs and enter the circulation to reach many tissues, and which can affect cellular gene expression of cancer cells and thereby their behaviour, survival, growth and invasiveness. Thus, influences such as WF inhalation affects the progression of many types of cancers, including those focused on in this study, specifically CC, PC, LC and GC, which are among the cancers most commonly linked with WF exposure^5–7^. The aim of this study is therefore to identify mechanisms through which WFs may increase cancer incidence.

LC is one of the most lethal types of cancer and globally is a leading cause of death^1,2,8^. WFs contain toxic metallic oxides and silicates that directly affect the sensitive tissues of the lung when inhaled, the manner of exposure (by inhalation) makes this the cancer with the highest risk for welders^9^. CC arises in the colon and the rectum and has a typical 5-year survival rate of about 60%. It damages colon or rectum by uncontrollable and invasive cell growth^10^. Iron, aluminum and magnesium oxide of the welding fumes are known to affect the incidence of CC^9^, although this is not well understood. PC affects prostate, the gland which produces seminal fluid and controls the transportation of sperm^11^. Nitrogen oxides, carbon dioxide and phosgene are risk factors for prostate neoplasms that are found in WFs^9^. GC (gastric or stomach cancer)^12^ is linked to exposures to nickel, beryllium and cobalt oxides which are all present in WFs^9^.

In this study, we developed a systematic and quantitative network-based approach to investigate the effects of WFs on gene expression and how these effects may give a clue as to how they encourage the incidence and progression of cancers through affecting pathways and pathway genes that are also altered in these cancers. Thus, we compared gene expression effects of WF exposure with the altered pattern of gene expression seen in CC, PC, LC and GC. This involved, firstly, analyzing differentially expressed gene profiles, then filtering these genes through gene-disease association networks, signaling and ontological pathways, and protein-protein interaction networks. We also investigated the importance of genes and pathways thus identified by using the gold benchmark databases dbGaP and OMIM to identify evidence to support the involvement of these genes in pathological processes such as cancer development. Moreover, we analysed patient survival and its association with the genes that are dysregulated in both the WF-exposed tissue and the four types of cancers. The influence on cancer patient survival of these identified genes provides evidence for their involvement in WF effect on cancer progression.

## Methods and Materials

### Overview of the analytical approach

**Figure 1:**
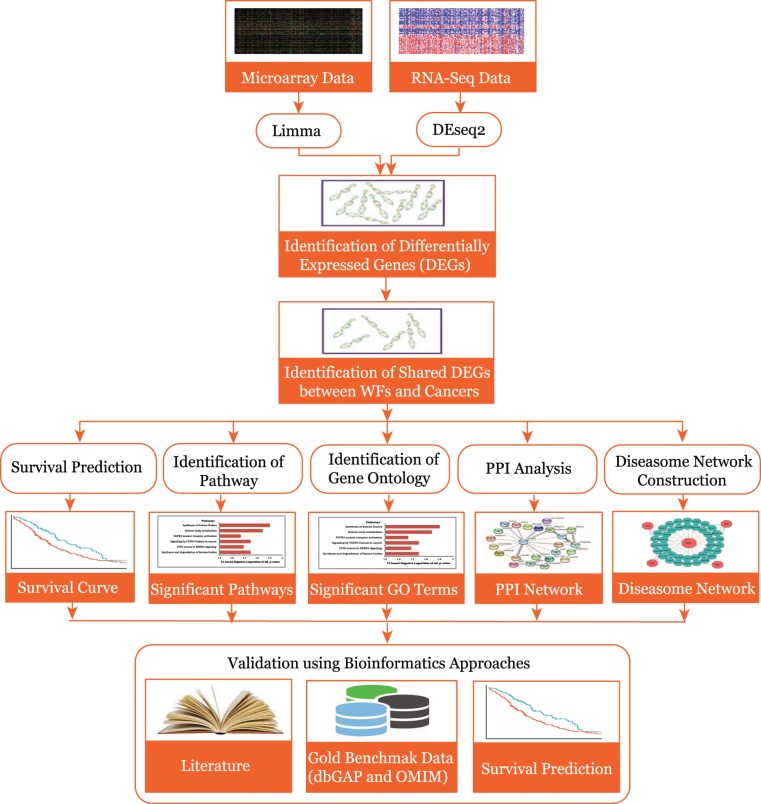
Flow-diagram of the analytical approach used in this study.

We applied an analytical approach to identify links between WF exposure and the incidence of the cancers by employing selected microarray datasets shown in the block diagram of the applied analytical approach shown in Fig. 1. This quantitative approach used genes differentially expressed in WF exposure, and identifies those that are also common to the differentially expressed genes observed in each cancer study. Further, these shared or common differentially expressed genes were used to construct gene-disease (diseasome) association network, identify signaling and ontological pathways, protein-protein interaction (PPI) network and survival function analysis. This approach also used gold benchmark databases OMIM and dbGaP validate genes and pathways identified in our study as showing possible disease associations.

### Datasets employed in this study

To identify the gene expression dysregulation that is common to WFs and the four types of cancers under investigation, we analyzed gene expression microarray datasets from the National Center for Biotechnology Information (NCBI). We examined five different microarray datasets with accession numbers GSE62384, GSE25071, GSE55945, GSE10072 and GSE2685^13–17^. Dataset GSE62384 was produced using human upper airway epithelial cells (RPMI 2650) exposed to spark generated WFs. These data were generated from cells exposed to WFs for 6 hours continuously at low (85 *μ*g/m^3^) and high (760 *μ*g/m^3^) concentrations. The CC dataset (GSE25071) consists of microarray data taken from 17 colorectal cancer sufferers who had late-onset CC (mean age 79 years) and 24 patients with early-onset CC (mean age 43 years). The PC dataset (GSE55945) is a microarray data on RNA taken from radical prostatectomy tissue from prostate cancer patients at the Beth Israel Deaconess Medical Center which compared tissue from PC sufferers (Gleason score 6 or 7) with normal prostate tissue. The LC dataset (GSE10072) contained microarray data comparing normal lung tissue and lung adenocarcinoma tissue collected from 26 former smokers, 20 non-smokers (who never smoked) and 28 current smokers; gene expression data are reported by comparing 49 non-tumor and 58 tumor lung tissues. The GC dataset (GSE2685) contains microarray data from 22 gastric cancer and 8 non-cancerous gastric tissues.

To analyze the patient survival association of the altered genes that are common to WFs and the four types of cancers under investigation, we retrieved clinical and RNAseq data for CC, PC, LC and GC from the cBioPortal ^18,19^. In the clinical dataset of CC (Colorectal Adenocarcinoma, TCGA, Nature 2012) there are 585 samples with 24 features. The samples of CC have RNAseq gene expression data included 224 cases with 224 mutated genes^20^. The clinical dataset of PC (Prostate Adenocarcinoma, TCGA, Cell 2015) includes 333 samples with 86 features. The RNAseq gene expression data of PC has 333 cases with 333 mutated genes^21^. The LC clinical dataset (Lung Adenocarcinoma, TCGA, PanCancer Atlas) consists of 566 samples with 81 features. The samples of LC have RNAseq gene expression data included 510 cases with 566 genes^22^. The clinical dataset of GC (Stomach Adenocarcinoma, TCGA, Nature 2014) contains 295 samples with 52 features. The samples of GC have RNAseq gene expression data included 265 cases with 295 mutated genes^23^. We employed six clinical factors (ethnicity, anatomical site of cancer, histological grade of cancer, primary tumour site, and neoplasm status with tumour) to analyze the survival of the altered genes that are common to WFs and the four types of cancers under investigation. The summarized description of the datasets is shown in Tables 1 and 2.

**Table 1:** Summarized description of the datasets used for gene expression and enrichment analysis.

Sl.	Disease name	GEO accession	Number of samples	
			Case	Healthy
1	Welding fumes (WFs)	GSE62384	18	06
2	Colorectal Cancer (CC)	GSE25071	46	04
3	Prostate Cancer (PC)	GSE55945	13	08
4	Lung cancer (LC)	GSE10072	58	49
5	Gastric Cancer (GC)	GSE2685	22	08

**Table 2:** Summarized description of the datasets used for survival prediction.

Sl.	Disease name	Datasets name in the cBioPortal	Number of samples			
			Patients	Clinical features	RNA-Seq	Mutated genes
2	Colorectal Cancer (CC)	Colorectal Adenocarcinoma (TCGA, Nature 2012)	585	24	224	224
3	Prostate Cancer (PC)	Prostate Adenocarcinoma (TCGA, Cell 2015)	333	86	333	333
4	Lung cancer (LC)	Lung Adenocarcinoma (TCGA, PanCancer Atlas)	566	81	510	566
5	Gastric Cancer (GC)	Stomach Adenocarcinoma (TCGA, Nature 2014)	295	52	265	295

### Analysis methods

Microarray-based gene expression analysis is a global and sensitive method to identify and quantify possible molecular mechanisms that underlie human disorders^24^. We used these approaches to analyze the gene expression profiles of CC, PC, LC and GC to find the genetic effects of WFs that may influence the development of these cancers. To allow comparisons of the mRNA expression data generated using different platforms and to avoid complications arising from the different experimental systems employed in the original studies, we normalized the gene expression data by means of Z-score transformation (*Z*_*ij*_) for each type of cancer tissue gene expression profile using ${Z}_{ij}=\frac{{g}_{ij}-mean({g}_{i})}{SD({g}_{i})}$, where *SD* denotes the standard deviation, *g*_*ij*_ denotes the value of the gene expression *i* in sample *j*. After this transformation gene expression values of different diseases at different platforms can be directly compared. We applied unpaired t-tests to find differentially expressed genes of each disease over control data and selected significantly dysregulated genes. We have chosen a threshold of at least 1 *log*_2_ fold change and a *p*-value for the t-tests of $< =1\times {10}^{-2}$. We employed the neighborhood-based benchmark and the multilayer topological methods to find gene-disease associations. We constructed a gene-disease network (GDN) using the gene-disease associations, where the nods in the network represent either gene or disease. This network can also be recognized as a bipartite graph. The primary condition for a disease to be connected with other diseases in GDN is they should share at least one or more significant dysregulated genes. Let $D$ is a specific set of diseases and *G* is a set of dysregulated genes, gene-disease associations attempt to find whether gene $g\in G$ is associated with disease $d\in D$. If ${G}_{i}$ and ${G}_{j}$, the sets of significantly dysregulated genes associated with diseases *D*_*i*_ and *D*_*j*_ respectively, then the number of shared dysregulated genes $({n}_{ij}^{g})$ associated with both disorders *D*_*i*_ and *D*_*j*_ is as follows^25^:

1 $${n}_{ij}^{g}=N({G}_{i}\cap {G}_{j})$$

The common neighbours are the based on the Jaccard Coefficient method, where the edge prediction score for the node pair is as^26^:

2 $$E(i,j)=\frac{N({G}_{i}\cap {G}_{j})}{N({G}_{i}\cup {G}_{j})}$$

where *G* is the set of nodes and *E* is the set of all edges. We used R software packages “comoR”^27^ and “POGO”^28^ to cross check their genes-disease associations.

To investigate how molecular determinants from the WF exposed tissues relate gene expression alterations in the cancers, we analyzed pathway and gene ontology using Enrichr ^29,30^. We used KEGG, WikiPathways, Reactome and BioCarta databases for analyzing signaling pathway^31–34^. We used GO Biological Process and Human Phenotype Ontology databases for ontological analysis^35,36^. We also constructed a protein-protein interaction sub-network for each CD, using the STRING database, a biological database and web resource of known and predicted protein-protein interactions^37^. Furthermore, we examined the validity of our study by employing two gold benchmark databases OMIM and dbGaP.

To determine the patient survival association of the altered genes that are common to WFs and the four types of cancers under investigation, we employed Cox PH model for univariate and multivariate analysis^38,39^. The Cox PH model can be written as follows:

3 $$h(t|{X}_{i})={h}_{0}(t)exp({\beta }^{T}{X}_{i})$$

Here $h(t|{X}_{i})$ is the hazard function conditioned on a subject $i$ with covariate information given as the vector ${X}_{i}$, ${h}_{0}(t)$ is the baseline hazard function which is independent of covariate information, and β represents a vector of regression coefficients to the covariates correspondingly. We have calculated the hazard ratio (HR) based on the estimated regression coefficients from the fitted Cox PH model to determine whether a specific covariate affects patient survival. The HR for a covariate ${x}_{r}$ can be expressed by the following simple formula exp $({\beta }_{r})$. Thus, the HR for any covariate can be calculated by applying an exponential function to the corresponding $({\beta }_{r})$ coefficient.

The survival status of a patient can be estimated by calculating PL estimator^40^ of the survival function can be defined as follows:

4 $$\hat{S}({t}_{j})=\mathop{\prod }\limits_{i=1}^{j}\,(1-\frac{{d}_{j}}{{n}_{j}})$$

Here $\hat{S}({t}_{j})$ is estimated survival function at time *t*_*j*_, *d*_*j*_ is the number of events occurred at *t*_*j*_, and *n*_*j*_ is the number of subjects available at *t*_*j*_. After estimating survival function, two or more groups can be compared using a log-rank test. We used Log-rank tests to detect the most significant genes in the case of patient’s survival time in altered versus normal (non-altered) groups in context of gene expression. The null hypothesis for this test can be symbolically explained as follows:

5 $${H}_{0}:{S}_{altered}(t)={S}_{normal}(t)$$

6 $${H}_{A}:{S}_{altered}(t)\ne {S}_{normal}(t)$$

Here *H*_0_ is survival functions that are the same for altered and normal gene and *H*_*A*_ is survival functions that are not the same for these two groups.

If the survival function of a specific gene is different among altered and normal groups then we include it to the combined Cox PH model. This approach is efficient for learning the effect of a specific gene of interest on patient survival in the presence of the clinical factors.

## Results

### Gene expression analysis

**Figure 2:**
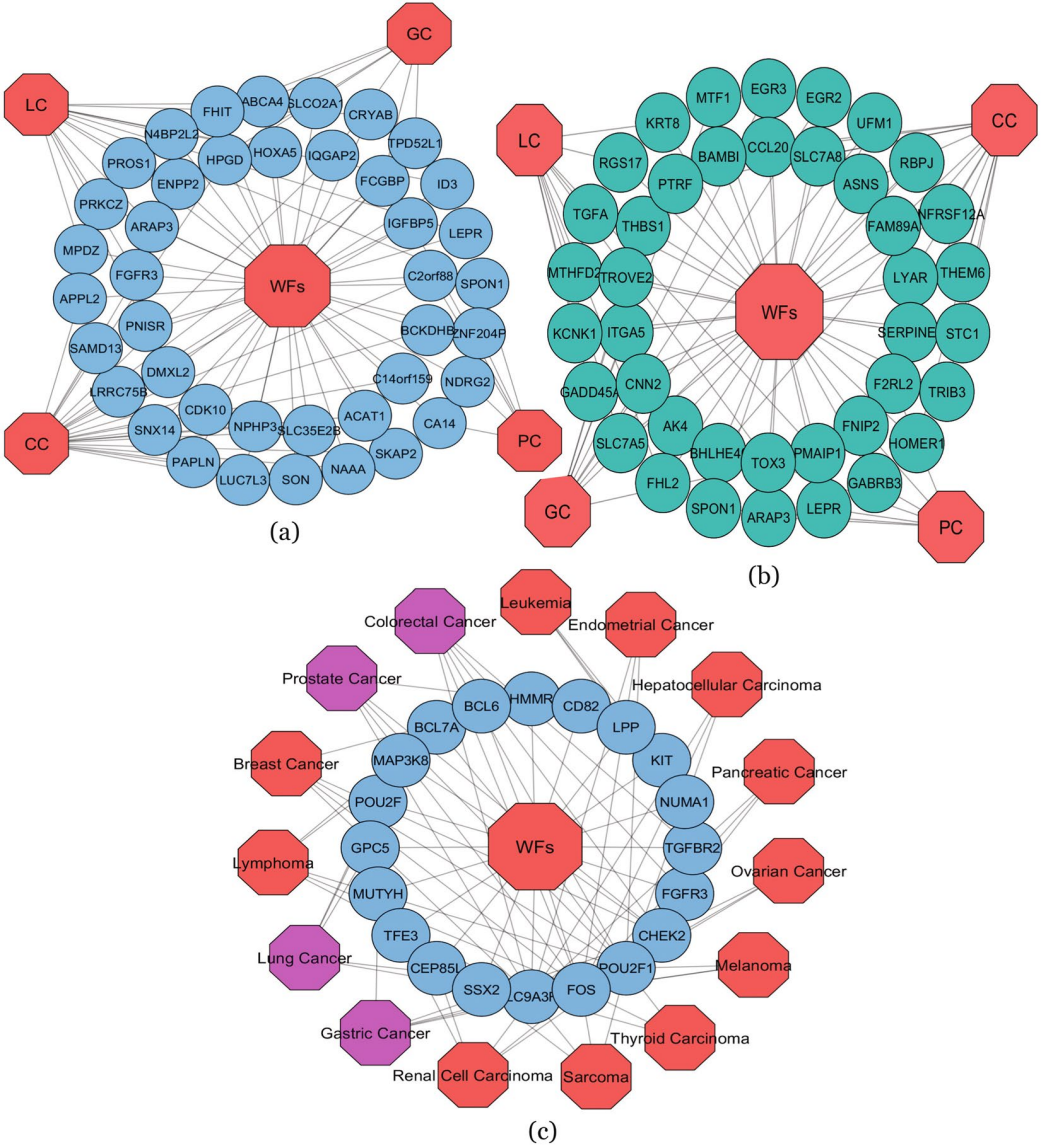
(**a**) Up-regulated gene-disease association network of welding fumes (WFs) exposure with colorectal cancer (CC), prostate cancer (PC), lung cancer (LC) and gastric cancer (GC). Octagon-shaped red-colored nodes represent different cancer types and sky-blue colored round-shaped nodes represent commonly up-regulated genes for WFs with the cancers examined. (**b**) Down-regulated gene-disease association network of welding fumes (WFs) exposure with colorectal cancer (CC), prostate cancer (PC), lung cancer (LC) and gastric cancer (GC). Octagon-shaped red colored nodes represent different cancer types and dark-cyan colored round-shaped nodes represent commonly down-regulated genes for WFs exposure with the different types of cancer examined. (**c**) Diseasome network showing validation of our study. Red colored octagon-shaped nodes represent different cancer types, pink-colored octagon-shaped nodes represent our selected four CDs and round-shaped sky-blue colored nodes represent differentially expressed genes for WFs exposure. A link is placed between a disease and a gene if mutations in that gene lead to the specific disease.

To identify and investigate the gene expression effects of WFs that may influence the behaviour of various types of cancer, we analyzed the gene expression microarray data collected from the National Center for Biotechnology Information (NCBI). We observed that WFs have 903 differentially expressed genes obtained by adjusted $p < =0.01$ and $|logFC| > =1$. The differentially expressed genes of WFs contain 392 up and 511 down-regulated genes relative to controls. Similarly, the statistical analysis identified the most significant genes with altered expression in each cancer type. The number of differentially expressed genes we identified was 939 (503 up and 436 down) in CC, 553 (323 up and 230 down) in PC, 890 (673 up and 217 down) in LC and 691 (463 up and 228 down) in GC. We also employed a cross-comparative analysis to find the common genes with altered expression between WFs and each CD. We found that WF treated cells share a number of differentially expressed genes with for CC (36 dysregulated genes), PC (13 genes), LC (25 genes) and GC (17 genes). To identify the significant associations among these cancer types with the effects of WF exposure, we constructed two separate gene-disease association-ship networks for up and down-regulated genes using Cytoscape plugins^41^, centered on the WF data as shown in Fig. 2(a,b). The necessary condition for two diseases to be associated is they must have at least one or more common differentially expressed genes in between them. Notably, two particular significant genes, C2orf88 and IGFBP5 were differentially expressed among WF exposure, CC and PC; and three significant genes, FCGBP, IQGAP2 and HPGD are common among WF exposure, CC and GC. One gene, FGFR3, is commonly dysregulated among WF exposure, CC and LC.

### Pathway and functional association analysis

**Figure 3:**
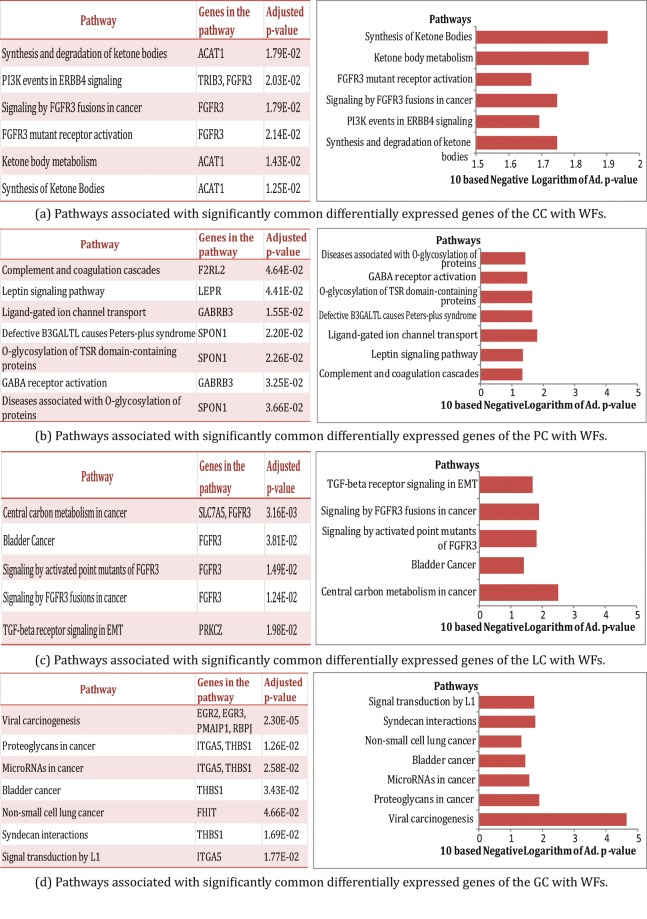
Pathway analysis for identifying the most significant signaling pathways common to the WF exposed cells and the cancer types revealed by the common differentially expressed genes. These include significant signaling pathways common to WFs exposed cells and (**a**) CC (**b**) PC (**c**) LC and (**d**) GC.

Pathways are constituted by a series of interactions at the molecular level in a cell, and are a vital key to understand the internal changes of an organism. Pathway-based analysis can be used to identify molecular or biological mechanisms that underlie the development of complex diseases^42,43^. We analyzed pathways of the commonly altered expression genes seen in WF exposure and in the cancers using Enrichr, a comprehensive web-based gene set enrichment analyzing tool^29,44^. Signaling pathways of the commonly altered expression genes of WF exposure and each type of cancer examined were analyzed using four globally recognized databases includes KEGG, WikiPathways, Reactome and BioCarta. We considered signaling pathways from the selected four databases and identified the most significant signaling pathways of each CD after applying several statistical analysis. Notably, we found 6, 7, 5 and 7 signaling pathways are associated with CC, PC, LC and GC, respectively, as shown in Fig. 3.

### Gene ontological analysis

The Gene Ontology (GO) refers to a universal conceptual model for representing gene functions and their relationship in the domain of gene regulation. It is constantly expanded by accumulating the biological knowledge to cover the regulation of gene functions and the relationship of these functions in terms of ontology classes and semantic relations between classes^45^. We analyzed ontological pathways of the commonly altered expression genes seen in WFs exposed cells and each cancer type using two recognized databases including GO Biological Process and Human Phenotype Ontology. We considered ontological pathways from selected two databases and identified the most significant ontological pathways for each cancer type after applying several statistical analysis. We found 10, 11, 14 and 14 ontological pathways are associated with the CC, PC, LC and GC, respectively, as shown in Tables 3–6.

**Table 3:** The most significant ontological pathways common to the WFs exposed cells and CC.

GO Term	Pathway	Genes in the pathway	Adjusted p-value
GO:0009083	Branched-chain amino acid catabolic process	BCKDHB, ACAT1	7.11E-04
GO:0009081	Branched-chain amino acid metabolic process	BCKDHB, ACAT1	7.78E-04
GO:0009063	Cellular amino acid catabolic process	BCKDHB, ACAT1	8.48E-04
GO:0051924	Regulation of calcium ion transport	HOMER1, STC1	4.10E-03
GO:0032092	Positive regulation of protein binding	BAMBI, TRIB3	5.39E-03
GO:0051726	Regulation of cell cycle	SON, HPGD, CDK10	6.82E-03
GO:0009966	Regulation of signal transduction	IGFBP5, HOMER1,FGFR3	8.49E-03
GO:0051099	Positive regulation of binding	BAMBI, TRIB3	9.56E-03
HP:0001643	Patent ductus arteriosus	HPGD, NPHP3, FGFR3	1.46E-03
HP:0001946	Ketosis	BCKDHB, ACAT1	1.16E-03

**Table 4:** The most significant ontological pathways common to the WFs exposed cells and PC.

GO Term	Pathway	Genes in the pathway	Adjusted p-value
GO:0071420	Cellular response to histamine	GABRB3	4.79E-03
GO:0044321	Response to leptin	LEPR	5.39E-03
GO:0035024	Negative regulation of Rho protein signal transduction	ARAP3	8.97E-03
GO:2000369	Regulation of clathrin-dependent endocytosis	ARAP3	9.56E-03
GO:0071417	Cellular response to organonitrogen compound	GABRB3, IGFBP5	1.52E-03
GO:2000146	Negative regulation of cell motility	IGFBP5, ARAP3	1.52E-03
GO:0030336	Negative regulation of cell migration	IGFBP5, ARAP3	2.34E-03
GO:0071407	Cellular response to organic cyclic compound	GABRB3, IGFBP5	2.90E-03
GO:0014912	Negative regulation of smooth muscle cell migration	IGFBP5	8.97E-03
HP:0000823	Delayed puberty	LEPR	1.67E-02
HP:0000824	Growth hormone deficiency	LEPR	1.67E-02

**Table 5:** The most significant ontological pathways common to the WFs exposed cells and LC.

GO Term	Pathway	Genes in the pathway	Adjusted p-value
GO:1903708	Positive regulation of hemopoiesis	N4BP2L2, HOXA5	4.18E-05
GO:0009132	Nucleoside diphosphate metabolic process	AK4	9.96E-03
GO:0045647	Negative regulation of erythrocyte differentiation	HOXA5	8.72E-03
GO:2000665	Regulation of interleukin-13 secretion	PRKCZ	8.72E-03
GO:0044320	Cellular response to leptin stimulus	LEPR	9.96E-03
GO:0045837	Negative regulation of membrane potential	PMAIP1	8.72E-03
GO:2000394	Positive regulation of lamellipodium morphogenesis	ENPP2	9.96E-03
GO:0050730	Regulation of peptidyl-tyrosine phosphorylation	ENPP2, PRKCZ	5.14E-03
GO:0032754	Positive regulation of interleukin-5 production	PRKCZ	9.96E-03
HP:0000975	Hyperhidrosis	SLCO2A1, FGFR3	5.38E-03
HP:0000522	Alacrima	FGFR3	1.24E-02
HP:0010662	Abnormality of the diencephalon	LEPR	1.86E-02
HP:0000495	Recurrent corneal erosions	FGFR3	1.49E-02
HP:0001413	Micronodular cirrhosis	KRT8	1.86E-02

**Table 6:** The most significant ontological pathways common to the WFs exposed cells and GC.

GO Term	Pathway	Genes in the pathway	Adjusted p-value
GO:0072331	Signal transduction by p53 class mediator	PMAIP1, FHIT	5.20E-04
GO:2001235	Positive regulation of apoptotic signaling pathway	PMAIP1, TPD52L1	1.92E-03
GO:0097193	Intrinsic apoptotic signaling pathway	PMAIP1, FHIT	3.33E-03
GO:0042981	Regulation of apoptotic process	UFM1, EGR3, PMAIP1, THBS1	4.28E-03
GO:0010634	Positive regulation of epithelial cell migration	ENPP2, THBS1	1.82E-03
GO:0045766	Positive regulation of angiogenesis	ITGA5, THBS1	3.46E-03
GO:0001936	Regulation of endothelial cell proliferation	EGR3, THBS1	2.07E-03
GO:0010038	Response to metal ion	MTF1, THBS1	1.72E-03
GO:0043066	Negative regulation of apoptotic process	UFM1, EGR3, ITGA5, THBS1	6.37E-04
GO:0051094	Positive regulation of developmental process	ENPP2, RBPJ	4.14E-03
GO:0034976	Response to endoplasmic reticulum stress	UFM1, THBS1	3.66E-03
GO:0043069	Negative regulation of programmed cell death	UFM1, EGR3, THBS1	4.66E-03
HP:0003577	Congenital onset	EGR2, HPGD	6.35E-03
HP:0000890	Long clavicles	HPGD	7.63E-03

### Protein-protein interaction analysis

**Figure 4:**
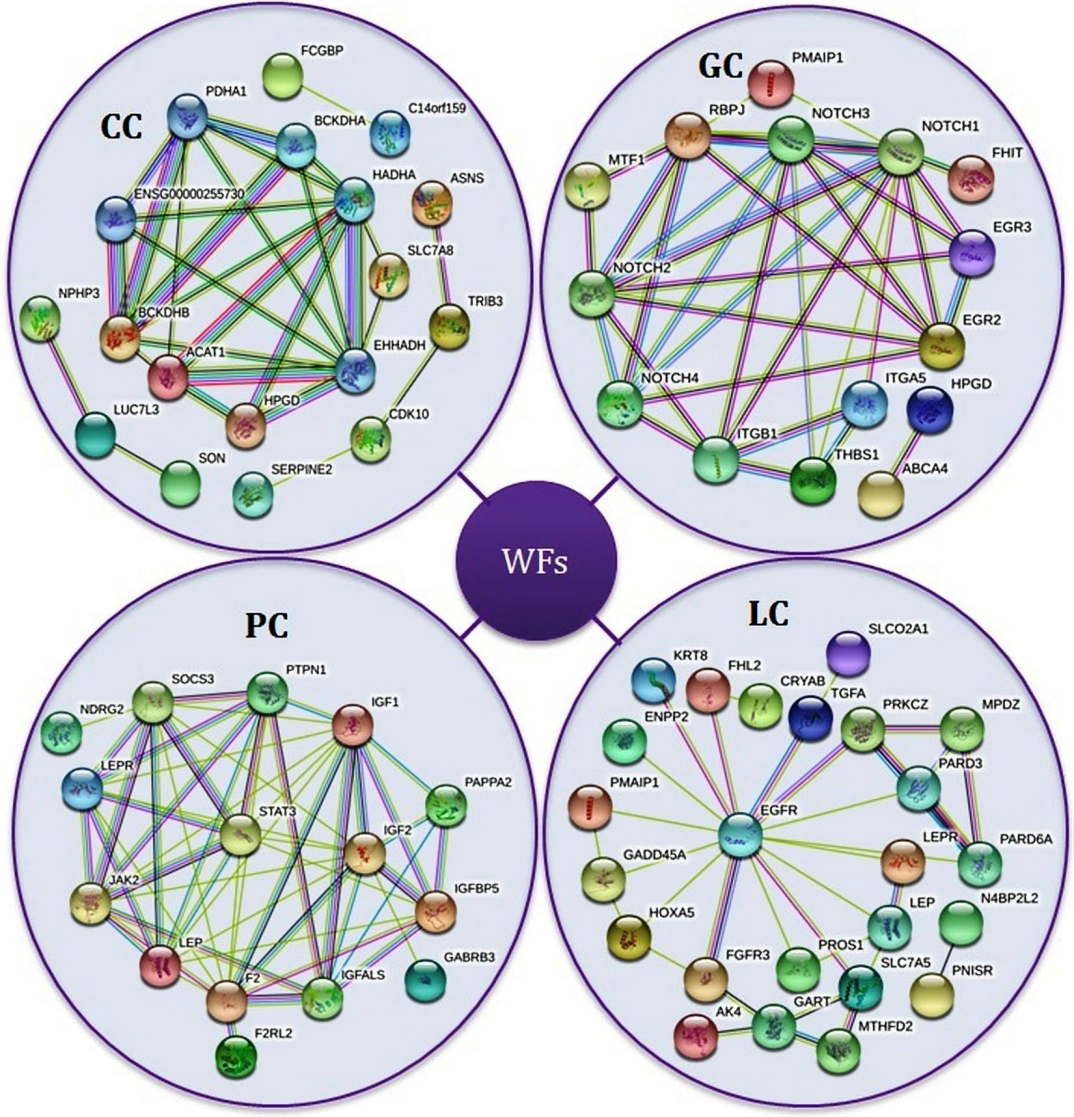
Protein-protein interaction network of the four types of cancer using STRING.

A protein-protein interaction network refers to the binding of proteins in the cell formed by biochemical or complex biological functions. Protein-protein interactions are essential to understand the cell physiology in health and disease states. We constructed and analyzed protein-protein interaction networks of the significantly altered expression genes of each CD using the STRING database. We clustered protein-protein interactions of cancer types into four different groups as shown in Fig. 4.

### Survival analysis

**Figure 5:**
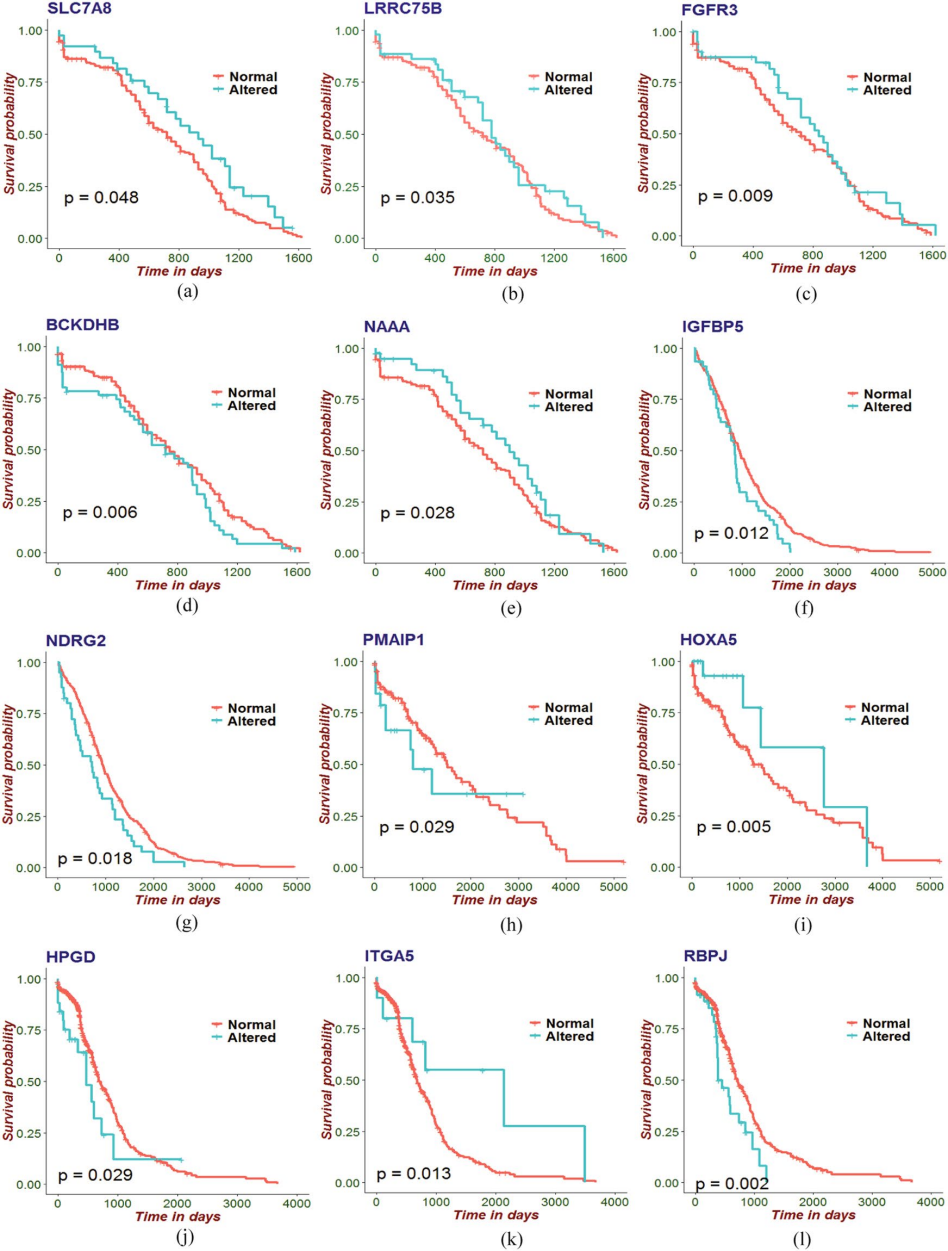
Survival function for an altered and normal group of the most significant genes that are common to WFs and the four types of cancers under investigation. These include significant genes common to WFs exposed cells and CC (**a–e**), PC (**f,g**), LC (**h,i**) and GC (**j–l**). Here, the cyan colored line in the survival graphs indicates the altered and the red indicates the normal gene expression group.

Patient survival analysis using both gene expression and clinical data is a popularly used feature in research to predict and characterize gene signatures in cancer^46^. In this study, we estimated survival function for altered and normal groups of the significant genes that are common to WFs and the four types of cancers under investigation by employing Cox PH model and PL estimator analysis. We fitted both univariate and multivariate analysis of the Cox PH regression model. The significant genes of the four selected cancers with estimated coefficients (*β*), hazard ratios (HR) and *p*-values from those analyses are shown in Tables 7–10. After these analyses we selected the most significant genes for the four types of cancers by choosing a threshold ($p < =0.05$) of the *p*-value. The survival curves of the most significant genes, comparing altered and normal groups had been obtained by using the PL estimator as shown in Fig. 5. Note that, from Fig. 5, we can see that those with altered expression of genes show lower survival compared to the normal group.

**Table 7:** *β* coefficient, hazard ratio and p-values in univariate, multivariate and combined models of the identified genes that are common between WFs and CC.

Gene symbol	Univariate			Multivariate			Combined		
	*β*	HR	p-value	*β*	HR	p-value	*β*	HR	p-value
NPHP3	−1.74E-01	8.40E-01	3.27E-01	−2.52E-01	7.77E-01	2.34E-01	−5.21E-02	9.49E-01	8.38E-01
SAMD13	8.58E-02	1.09E+00	6.66E-01	1.24E-02	1.01E+00	9.58E-01	1.97E-01	1.22E+00	4.55E-01
STC1	9.02E-02	1.09E+00	6.19E-01	1.08E-01	1.11E+00	6.06E-01	−1.08E-01	8.98E-01	6.56E-01
THEM6	9.20E-02	1.10E+00	5.89E-01	8.11E-02	1.08E+00	6.97E-01	2.95E-01	1.34E+00	2.10E-01
TNFRSF12A	3.16E-01	1.37E+00	8.94E-02	6.14E-01	1.85E+00	5.27E-03	8.19E-01	2.27E+00	6.48E-01
ASNS	1.35E-01	1.14E+00	5.23E-01	1.66E-01	1.18E+00	4.86E-01	2.38E-01	1.27E+00	4.17E-01
FAM89A	2.19E-01	1.24E+00	2.12E-01	3.28E-01	1.39E+00	1.12E-01	1.80E-01	1.20E+00	4.13E-01
ACAT1	−3.24E-02	9.68E-01	8.49E-01	−9.12E-02	9.13E-01	6.55E-01	1.41E-01	1.15E+00	5.78E-01
HPGD	3.90E-01	1.48E+00	6.10E-02	3.63E-01	1.44E+00	1.25E-01	2.60E-01	1.30E+00	3.36E-01
**SLC7A8**	−**3.99E**−**01**	**6.71E-01**	**5.01E-02**	−**2.38E-01**	**7.88E-01**	**3.46E-01**	−**5.80E-01**	**5.60E-01**	**4.87E-02**
TRIB3	1.30E-02	1.01E+00	9.39E-01	−9.81E-02	9.07E-01	6.12E-01	−3.09E-01	7.34E-01	1.58E-01
APPL2	−1.63E-01	8.50E-01	4.50E-01	−1.29E-01	8.79E-01	5.98E-01	5.43E-02	1.06E+00	8.45E-01
**LRRC75B**	−**1.42E-01**	**8.67E-01**	**4.52E-01**	−**3.28E-01**	**7.20E-01**	**1.35E-01**	−**5.23E-01**	**5.93E-01**	**3.58E-02**
BAMBI	4.46E-01	1.56E+00	1.39E-02	5.17E-01	1.68E+00	1.53E-02	2.39E-01	1.27E+00	3.32E-01
**FGFR3**	−**2.06E-01**	**8.14E-01**	**3.04E-01**	−**6.86E-01**	**5.03E-01**	**4.39E-03**	−**8.88E-01**	**4.11E-01**	**9.54E-03**
DMXL2	5.36E-02	1.06E+00	7.46E-01	3.93E-02	1.04E+00	8.45E-01	1.93E-01	1.21E+00	3.98E-01
CCL20	2.16E-01	1.24E+00	2.62E-01	4.86E-02	1.05E+00	8.24E-01	1.03E-02	1.01E+00	9.67E-01
SERPINE2	−3.53E-01	7.03E-01	6.55E-02	−4.57E-01	6.33E-01	4.42E-02	−5.14E-01	5.98E-01	4.31E-01
CDK10	3.49E-02	1.04E+00	8.50E-01	1.02E-01	1.11E+00	6.21E-01	4.02E-01	1.50E+00	9.56E-02
SON	4.35E-01	1.54E+00	1.48E-02	2.89E-01	1.33E+00	1.44E-01	3.43E-01	1.41E+00	1.78E-01
FCGBP	1.18E-01	1.12E+00	5.39E-01	2.44E-01	1.28E+00	2.91E-01	2.33E-01	1.26E+00	3.44E-01
IQGAP2	−6.58E-02	9.36E-01	7.27E-01	1.66E-01	1.18E+00	4.71E-01	2.61E-01	1.30E+00	2.94E-01
LUC7L3	−9.57E-03	9.90E-01	9.57E-01	−2.53E-01	7.77E-01	2.32E-01	−2.38E-02	9.77E-01	9.22E-01
**BCKDHB**	**2.77E-01**	**1.32E+00**	**1.01E-01**	**4.12E-01**	**1.51E+00**	**3.43E-02**	**5.91E-01**	**1.81E+00**	**6.52E-03**
LYAR	2.14E-01	1.24E+00	2.71E-01	1.13E-01	1.12E+00	6.14E-01	−1.11E-01	8.95E-01	6.57E-01
**NAAA**	−**2.33E-01**	**7.92E-01**	**2.44E-01**	−**2.19E-01**	**8.03E-01**	**4.02E-01**	−**5.92E-01**	**5.53E-01**	**2.86E-02**
SKAP2	−1.80E-01	8.35E-01	3.12E-01	−2.83E-01	7.54E-01	1.70E-01	−1.09E-01	8.97E-01	6.46E-01
BHLHE40	4.44E-03	1.00E+00	9.81E-01	−1.13E-01	8.94E-01	6.30E-01	−1.50E-01	8.61E-01	5.75E-01
IGFBP5	−4.24E-02	9.59E-01	8.31E-01	9.18E-02	1.10E+00	6.98E-01	2.18E-01	1.24E+00	3.99E-01
SNX14	2.05E-02	1.02E+00	9.08E-01	6.87E-02	1.07E+00	7.37E-01	−1.35E-01	8.74E-01	5.59E-01
HOMER1	−1.84E-01	8.32E-01	3.60E-01	−2.37E-01	7.89E-01	2.83E-01	−3.02E-01	7.39E-01	2.03E-01
PAPLN	−3.29E-01	7.20E-01	1.23E-01	−4.48E-01	6.39E-01	8.79E-02	−2.94E-01	7.45E-01	3.21E-01

**Table 8:** *β* coefficient, hazard ratio and p-values in univariate, multivariate and combined models of the identified genes that are common between WFs and PC.

Gene symbol	Univariate			Multivariate			Combined		
	β	HR	p-value	β	HR	p-value	β	HR	p-value
ARAP3	1.12E-01	1.12E+00	4.48E-01	1.81E-01	1.20E+00	2.57E-01	2.38E-01	1.27E+00	1.39E-01
CA14	1.49E-01	1.16E+00	3.74E-01	1.64E-01	1.18E+00	3.59E-01	2.11E-01	1.24E+00	2.45E-01
F2RL2	−4.70E-02	9.54E-01	7.53E-01	−8.16E-02	9.22E-01	6.00E-01	−5.31E-02	9.48E-01	7.41E-01
FNIP2	3.27E-03	1.00E+00	9.84E-01	−6.86E-03	9.93E-01	9.68E-01	1.20E-01	1.13E+00	4.93E-01
GABRB3	−9.83E-02	9.06E-01	4.13E-01	−1.21E-01	8.86E-01	3.28E-01	−1.14E-01	8.93E-01	3.67E-01
**IGFBP5**	**3.63E-01**	**1.44E+00**	**2.28E-02**	**3.85E-01**	**1.47E+00**	**1.86E-02**	**4.17E-01**	**1.52E+00**	**1.23E-02**
LEPR	−1.95E-02	9.81E-01	8.78E-01	−2.36E-02	9.77E-01	8.57E-01	−9.40E-02	9.10E-01	4.84E-01
**NDRG2**	**4.03E-01**	**1.50E+00**	**1.61E-02**	**3.80E-01**	**1.46E+00**	**2.74E-02**	**4.16E-01**	**1.52E+00**	**1.85E-02**
SPON1	−8.50E-02	9.19E-01	6.38E-01	−2.19E-01	8.04E-01	2.66E-01	−3.41E-01	7.11E-01	8.93E-02
TOX3	−2.11E-02	9.79E-01	8.95E-01	8.41E-03	1.01E+00	9.60E-01	−5.66E-02	9.45E-01	7.40E-01

**Table 9:** *β* coefficient, hazard ratio and p-values in univariate, multivariate and combined models of the identified genes that are common between WFs and LC.

Gene symbol	Univariate			Multivariate			Combined		
	β	HR	p-value	β	HR	p-value	β	HR	p-value
PROS1	−5.57E-02	9.46E-01	8.12E-01	2.56E-02	1.03E+00	9.28E-01	2.42E-01	1.27E+00	4.09E-01
SPON1	1.76E-01	1.19E+00	5.24E-01	4.52E-01	1.57E+00	1.72E-01	4.30E-01	1.54E+00	2.48E-01
KCNK1	−2.05E-01	8.15E-01	5.02E-01	−1.35E-01	8.73E-01	7.04E-01	−2.63E-01	7.68E-01	4.76E-01
KRT8	1.19E-01	1.13E+00	6.81E-01	2.11E-01	1.23E+00	5.35E-01	2.88E-01	1.33E+00	4.21E-01
ID3	7.21E-02	1.07E+00	8.34E-01	1.62E-01	1.18E+00	7.43E-01	1.13E-01	1.12E+00	8.39E-01
CRYAB	−1.58E-01	8.54E-01	5.87E-01	−8.98E-03	9.91E-01	9.79E-01	−1.44E-01	8.66E-01	6.85E-01
FGFR3	4.95E-01	1.64E+00	1.49E-01	5.82E-01	1.79E+00	1.71E-01	4.73E-01	1.60E+00	2.80E-01
ENPP2	−3.64E-01	6.95E-01	1.99E-01	−5.45E-01	5.80E-01	1.26E-01	−5.27E-01	5.90E-01	1.44E-01
**PMAIP1**	**2.78E-01**	**1.32E+00**	**4.37E-01**	**6.49E-01**	**1.91E+00**	**1.31E-01**	**1.02E+00**	**2.78E+00**	**2.91E-02**
RGS17	−1.95E-01	8.23E-01	4.77E-01	−2.73E-01	7.61E-01	4.00E-01	−4.14E-02	9.59E-01	8.99E-01
MTHFD2	2.14E-01	1.24E+00	4.29E-01	4.27E-01	1.53E+00	2.89E-01	5.09E-01	1.66E+00	2.13E-01
ARAP3	3.37E-01	1.40E+00	2.32E-01	3.25E-01	1.38E+00	3.89E-01	5.24E-01	1.69E+00	1.83E-01
TGFA	3.10E-01	1.36E+00	3.66E-01	3.53E-01	1.42E+00	4.32E-01	4.43E-01	1.56E+00	3.62E-01
**HOXA5**	**−6.82E-01**	**5.06E-01**	**1.41E-01**	**−1.59E+00**	**2.03E-01**	**9.55E-03**	**−1.86E+00**	**1.56E-01**	**5.18E-03**
PNISR	−1.38E-01	8.71E-01	6.23E-01	−6.86E-01	7.56E-01	4.19E-01	−4.15E-01	6.60E-01	2.44E-01
GADD45A	2.66E-01	1.31E+00	3.07E-01	−2.80E-01	1.07E+00	8.28E-01	2.42E-01	1.27E+00	4.78E-01
FHL2	−3.39E-02	9.67E-01	9.02E-01	7.20E-02	6.50E-01	2.89E-01	−2.87E-01	7.51E-01	4.61E-01
TOX3	−1.10E-01	8.96E-01	6.91E-01	−4.30E-01	5.89E-01	1.65E-01	−7.57E-01	4.69E-01	6.12E-02
LEPR	1.51E-01	1.16E+00	5.71E-01	−5.29E-01	1.12E+00	7.35E-01	2.02E-01	1.22E+00	5.45E-01
SLCO2A1	−2.08E-01	8.13E-01	5.45E-01	1.12E-01	8.36E-01	6.82E-01	−1.20E-01	8.87E-01	7.90E-01
SLC7A5	1.42E-01	1.15E+00	5.88E-01	−1.79E-01	1.23E+00	5.05E-01	−2.26E-02	9.78E-01	9.44E-01
MPDZ	2.37E-01	1.27E+00	3.00E-01	2.11E-01	1.34E+00	2.58E-01	4.03E-01	1.50E+00	1.42E-01

**Table 10:** *β* coefficient, hazard ratio and p-values in univariate, multivariate and combined models of the identified genes that are common between WFs and GC.

Gene symbol	Univariate			Multivariate			Combined		
	β	HR	p-value	β	HR	p-value	β	HR	p-value
ABCA4	1.44E-01	1.16E+00	6.89E-01	2.86E-01	1.33E+00	4.53E-01	3.51E-01	1.42E+00	3.60E-01
CNN2	−9.03E-02	9.14E-01	6.45E-01	−2.10E-01	8.10E-01	3.12E-01	−3.64E-01	6.95E-01	9.73E-02
EGR2	−2.19E-01	8.03E-01	6.64E-01	−9.34E-02	9.11E-01	8.58E-01	−7.03E-02	9.32E-01	8.96E-01
EGR3	−2.51E-01	7.78E-01	4.41E-01	−1.95E-01	8.23E-01	5.64E-01	−5.92E-02	9.43E-01	8.65E-01
ENPP2	−6.84E-02	9.34E-01	8.04E-01	−6.85E-02	9.34E-01	8.10E-01	6.74E-02	1.07E+00	8.20E-01
FCGBP	−5.25E-02	9.49E-01	8.66E-01	−2.88E-02	9.72E-01	9.29E-01	−6.37E-03	9.94E-01	9.84E-01
FHIT	−2.65E-02	9.74E-01	9.38E-01	3.03E-01	1.35E+00	3.98E-01	3.37E-01	1.40E+00	3.51E-01
**HPGD**	**4.71E-01**	**1.60E+00**	**8.91E-02**	**6.97E-01**	**2.01E+00**	**2.22E-02**	**6.83E-01**	**1.98E+00**	**2.90E-02**
IQGAP2	−4.14E-01	6.61E-01	1.82E-01	−7.08E-01	4.93E-01	3.29E-02	−6.01E-01	5.48E-01	7.69E-02
**ITGA5**	**−8.11E-01**	**4.45E-01**	**5.39E-02**	**−9.77E-01**	**3.77E-01**	**2.95E-02**	**−1.16E+00**	**3.15E-01**	**1.35E-02**
MTF1	−9.49E-02	9.10E-01	6.40E-01	−1.21E-01	8.86E-01	5.76E-01	−6.43E-02	9.38E-01	7.71E-01
PMAIP1	−5.46E-02	9.47E-01	8.44E-01	−1.10E-01	8.96E-01	7.18E-01	−6.08E-02	9.41E-01	8.45E-01
**RBPJ**	**5.92E-01**	**1.81E+00**	**6.64E-03**	**7.14E-01**	**2.04E+00**	**2.41E-03**	**7.31E-01**	**2.08E+00**	**2.37E-03**
THBS1	−2.71E-01	7.63E-01	4.52E-01	−2.75E-01	7.60E-01	4.62E-01	−2.04E-01	8.15E-01	5.85E-01
TPD52L1	2.90E-03	1.00E+00	9.92E-01	−1.43E-01	8.67E-01	6.54E-01	−1.79E-01	8.36E-01	5.80E-01
TROVE2	−1.26E-02	9.88E-01	9.54E-01	−1.33E-01	8.75E-01	5.78E-01	−1.54E-01	8.57E-01	5.25E-01
UFM1	−1.47E-01	8.63E-01	3.50E-01	−1.61E-01	8.51E-01	3.52E-01	−2.06E-01	8.14E-01	2.46E-01

## Discussion

In this study we investigated how WF exposure may influence a number of types of cancer whose development and growth is greater with exposure to WFs or the components of WFs. We compared the gene expression alterations that result from WF exposure in cells with that of the genes that have dysregulated expression in several cancer types. The idea behind this is similar to studies of comorbidities, where dysregulated genes (or more usually gene pathways) that are common to two diseases give clues to how those diseases interact when co-occurring in the same individual, even if we are unclear as to the reason for the altered expression of individual genes or pathways is unclear. Thus, genes or gene pathways altered in response to WF exposure and the cancers of interest can be means by which WF exposure encourages those cancers to develop. Note that WFs included components such as metal fumes that are absorbed by the lungs into the bloodstream, to expose many tissues around the body. Many of these fumes are carcinogenic, but cancer initiation is only one of a number of stages of cancer development and progression, and welders commonly have regular exposure to fumes over long periods. Unlike in other morbidities, some altered gene expression may arise in individual cancer cells due to mutations which will affect survival of those cells; if such altered expression the is detected in whole cancer tissue across many individuals (as in our studies) then the alteration may be affecting pathways that encourage survival and growth. Thus we have applied a systematic approach to identify pathways that WFs may affect the cancer behaviours.

For our analysis we employed gene regulation analysis, gene-disease association networks, signaling and ontological pathways, and protein-protein interaction networks. To identify pathways and genes that are important in WF interactions in the cellular processes that influence cancer progression, we examined gene expression microarray data from WF exposed cells, CC, PC, LC and GC, each with control datasets. This identified a large number of significant genes that were commonly dysregulated between WF-exposure and cancer profiles, and evident by simple gene expression comparisons. There were a number of dysregulated genes that were common between WF exposure responses and cancer types, which suggests that WF exposure may cause gene expression changes that could affect the behaviour of cancers. It should be noted that the cancer transcriptome datasets, such as those employed here, contain transcripts from both cancer cells and the supporting stromal cells found in the tumors themselves. Thus, it should be noted that WFs may exert their effects on cancers either indirectly (through tumor stroma) or on the cancer cells themselves.

We constructed two separate gene-disease association networks for up- and down-regulated genes showed strong evidence that WFs may indeed influence these cancers as indicated in Fig. 2(a,b). The pathway-based analysis is a technique to better understand the molecular or biological mechanisms underlying different complex diseases by determining common pathways that a stimulus (such as WFs) may influence cells of interest. We identified significant signaling and ontological pathways of the commonly dysregulated genes of each cancer. These identified pathways indicated how WFs may affect these cancer types. Similarly, protein-protein interaction sub-networks of the commonly altered genes suggest that WFs affect several types of cancers. Note that if a pathway is a conduit for the effects of an important risk factor for a disease, this points to that pathway being particularly important to the pathogenesis of the disease and that reducing that pathways effects could be a way to attack the disease progression itself. It should be noted that these findings only point to possible ways that WF exposure may affect the cancers and cannot prove causation. However, when we investigated whether the gene expression patterns that we have observed could be associated with reduced survival of the patients (pointing to the importance of those gene expression levels either directly or indirectly) that is what we observed for several of significant genes that are common WF the cancer profiles under investigation as shown in Fig. 5.

It should be noted that the datasets employ a number of different cell types, which is commonly the case in this type of study. While gene expression patterns are, by definition, different in different cell types, here we were only concerned with expression alterations; certain responses to WFs may not occur in all cells so, while our approach cannot identify all pathways affected by WFs in nascent tumour cells, it will find some. Indeed, our data provides evidence to suggest the involvement of a number of genes in cancer behaviours that are linked to the noxious effect of WFs on cancer.

We used the gold benchmark databases OMIM and dbGaP for cross checking the validity of our outcome and found that there were some shared genes in between the WF exposure and cancer types as shown in Fig. 2(c). For validation purposes, we collected disease with associated genes from the dbGaP, OMIM Disease and OMIM Expanded databases using differentially expressed genes of WFs. After several steps of statistical analysis we selected only cancer related diseases. Interestingly, we found our selected four cancers among the list of cancers collected from the mentioned databases as shown in Fig. 2(c).

Moreover, we found our identified genes in Fig. 2(c) had been shown in other studies to be associated with disease progression in cancers. Specifically, VÃ¡zquez-ArreguÃn K. *et al*., Cybulski C. *et al*. and Wang L. *et al*. shown RAB4B, CHEK2 and FOS to be associated with CC incidence^47–50^; Biswas S. *et al*. found a link between TGFBR2 and CC^51^. Lijovic M. *et al*. showed CD82 to be linked to PC incidence^52^; Wang Y. *et al*. shown the association between CHEK2 and PC progression^53^; Ouyang X. *et al*. identified a link between FOS and PC^54^; Gruosso T. *et al*. showed MAP3K8 to be associated with LC^55^; Vallejo A. *et al*. found a link between FOS and LC incidence^56^; Yuan S. *et al*. showed an association between GPC5 and LC progression^57^. Kim CJ. *et al*. found MUTYH to be associated to GC incidence^58^; Myllykangas S. found an association between FOS and GC^59^; Teodorczyk U. *et al*. found CHEK2 to be linked to GC progression^60^. Therefore, it suggested that WFs may have a strong interaction with CC, PC, LC and GC.

## Conclusions

In this study, we considered gene expression microarray data from WFs exposure, CC, PC, LC, GC and control datasets to analyze and investigate the genetic links between WF exposure and the effects that they have on cancers. We analyzed gene expression, constructed gene-disease association networks, identified signaling and ontological pathways, analyzed protein-protein interaction networks and survival function of WFs exposed cells and cancers. The outcome of our study indicated that WFs can exert a strong influence on cancers. This kind of study will be useful for making more accurate disease prediction, and identifyi potentially better therapeutic approaches. This study will also be useful for assessing the dangerous effects of welding on the human body.
